# Nanotechnology for Topical Drug Delivery to the Anterior Segment of the Eye

**DOI:** 10.3390/ijms222212368

**Published:** 2021-11-16

**Authors:** Alexander Vaneev, Victoria Tikhomirova, Natalia Chesnokova, Ekaterina Popova, Olga Beznos, Olga Kost, Natalia Klyachko

**Affiliations:** 1 Chemistry Faculty, M.V. Lomonosov Moscow State University, 119991 Moscow, Russia; vaneev.aleksandr@gmail.com (A.V.); vetikhomirova@gmail.com (V.T.); popova.ekaterina1995@gmail.com (E.P.); olga.a.kost@gmail.com (O.K.); 2 Research Laboratory of Biophysics, National University of Science and Technology “MISIS”, 119991 Moscow, Russia; 3 Department of Pathophysiology and Biochemistry, Helmholtz National Medical Research Center of Eye Diseases, 105062 Moscow, Russia; nchesnokova2012@yandex.ru (N.C.); olval2011@mail.ru (O.B.); 4 Eshelman School of Pharmacy, University of North Carolina at Chapel Hill, Chapel Hill, NC 27599, USA; 5 Research Institute “Nanotechnology and Nanomaterials”, G.R. Derzhavin Tambov State University, 392000 Tambov, Russia

**Keywords:** ocular drug delivery, anterior segment of the eye, nanoparticles, nanomicelles, in situ gels, ocular barriers

## Abstract

Topical drug delivery is one of the most challenging aspects of eye therapy. Eye drops are the most prevalent drug form, especially for widely distributed anterior segment eye diseases (cataracts, glaucoma, dry eye syndrome, inflammatory diseases, etc.), because they are convenient and easy to apply by patients. However, conventional drug formulations are usually characterized by short retention time in the tear film, insufficient contact with epithelium, fast elimination, and difficulties in overcoming ocular tissue barriers. Not more than 5% of the total drug dose administered in eye drops reaches the interior ocular tissues. To overcome the ocular drug delivery barriers and improve drug bioavailability, various conventional and novel drug delivery systems have been developed. Among these, nanosize carriers are the most attractive. The review is focused on the different drug carriers, such as synthetic and natural polymers, as well as inorganic carriers, with special attention to nanoparticles and nanomicelles. Studies in vitro and in vivo have demonstrated that new formulations could help to improve the bioavailability of the drugs, provide sustained drug release, enhance and prolong their therapeutic action. Promising results were obtained with drug-loaded nanoparticles included in in situ gel.

## 1. Introduction

According to the World Health Organization data, about 300 million people are living with serious vision disorders worldwide. The number of people who live with some form of distance or near vision impairment is about 2.2 billion, among them 33.6 million people are blind, while in at least 1 billion cases vision impairment could have been prevented [1].

**Figure 1:**
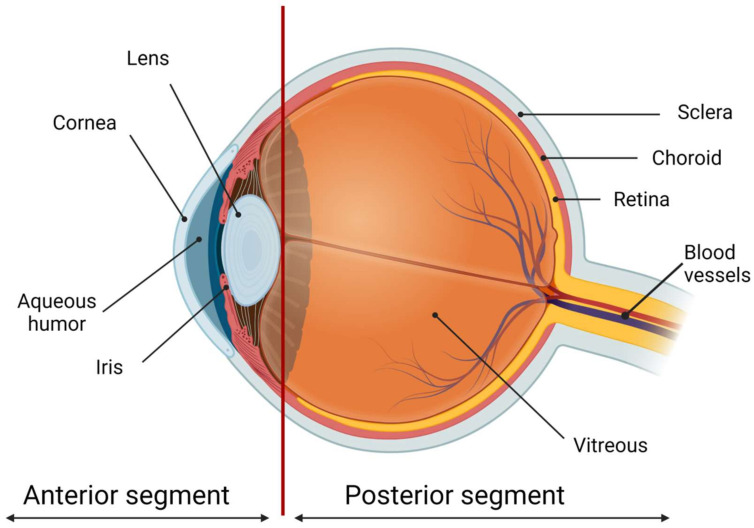
The scheme of the eye. The ocular globe can be conditionally divided into two parts: the anterior segment and the posterior segment. The anterior eye segment consists of the cornea, conjunctiva, iris, ciliary body, lens, and aqueous humor, while the sclera, choroid, retina, and vitreous body form the posterior segment. Created with BioRender.com.

Most of the patients with eye diseases receive drug therapy. The bioavailability of the ophthalmic drugs after systemic administration is strongly restricted by the blood-ocular barrier system formed by two main barriers: the blood-aqueous barrier for the anterior segment and the blood-retinal barrier for the posterior eye segment (Figure 1). The former consists of the tight junctions of the non-pigmented epithelium of the ciliary body, iris tissues, and the iris blood vessels. This barrier limits the drug access to the anterior segment of the eye. The blood-retinal barrier is formed by junctions between retinal capillary endothelial cells and the tight junctions between retinal pigment epithelial cells [2].

The required amount of eye medication by local administration is significantly less than that by systemic administration. Local injections (subconjunctival or intravitreal) of the drugs are traumatic and have to be performed by medical personnel only. Topical administration of eye medications in the form of eye drops or gels is more favorable, as it can be easily performed by patients themselves. Eye drops are the most prevalent way of therapy for anterior eye segment diseases because they are convenient, demand less amount of the drug, and, consequently, are much less likely to cause side effects than after systemic administration. It is the major delivery route used for optimal drug absorption, especially for the treatment of anterior eye segment diseases. Eye drops represent about 90% of the marketed ophthalmic formulations [3,4,5,6,7,8].

However, only not more than 5% of the total dispensed dose of the drug administered in eye drops reaches the interior ocular tissues [9]. Only ~30 µL of the liquid drug formulation can be applied to the eye due to the limited ocular surface area, and most of it is rapidly eliminated from the eye surface due to the tear turnover, blinking, and nasolacrimal drainage. Two minutes after instillation, about 60% of the drug was reported to be eliminated; after 8 min, the drug was diluted at 1/1000 and after 15–25 min, almost all an active ingredient was eliminated from the eye surface [10].

**Figure 2:**
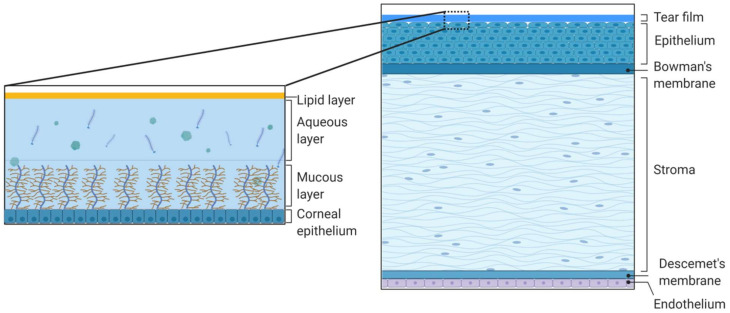
Schematic representation of the structure and composition of the cornea and the tear film. The cornea consists of the tear film, epithelium, Bowman’s membrane, stroma, Descemet’s membrane, and inner endothelium. The tear film consists of the outer lipid phase, intermediate aqueous phase, and mucus. Created with BioRender.com.

Besides, tissue and fluid eye barriers restrict the influx of the active substance into the inner eye structures. The main route of topically administered agents into the anterior segment of the eye is via absorption through the cornea and conjunctiva with the subsequent transfer into the anterior chamber. The tear film on the cornea and conjunctiva covering the rest eye surface and the interior surface of the eyelids is the first permeability barrier limiting intraocular drug delivery. Tear film covers, hydrates, and protects the ocular surface, provides the eyelids sliding, and may delay the penetration of drugs into the eye. The human conjunctival sac can transitorily contain about 30 μL of tear fluid, and normal tear volume is estimated at roughly 7 μL. Tears have a high turnover rate (restoration time of 2–3 min), thus limiting ocular residence time for a drug [11]. The tear film is a thin transparent fluid layer composed of three phases including an outer lipid phase, an intermediate aqueous phase, and an inner mucin layer (Figure 2). The external lipid layer is 0.1 microns thick and prevents the evaporation of the tear film, as well as prevents the tear overflow. The 7–8 microns aqueous layer is the main component of the tear film. Proteins and enzymes in the aqueous phase can bind and metabolize the active drug, leading to the decrease of ocular drug bioavailability. The mucin inner layer has a thickness of 0.2 microns. Mucins of the tear film are heavily glycosylated proteins (glycoconjugates). High sialic acid and sulfate content in most mucins provide a net-negative surface charge of the eye [12]. Thus, mucins can attract or repulse drugs via electrostatic interactions depending on the charge of the drug molecule or carrier system [5,13]. The osmolarity of the tear fluid is close to that of the blood, however, tears also contain weak buffering systems in the form of carbonate ions and weak organic acids, which can change the extent of drug ionization and, therefore, its bioavailability [11].

The bulbar conjunctiva is a mucous membrane that covers the sclera, and the palpebral conjunctiva lines the inner side of the eyelids. Conjunctiva consists of the surface epithelium and inner stroma layer that comprise connective tissues with blood and lymphatic vessels. Conjunctiva is more permeable than the cornea, and its surface area is about 17 times larger than that of the cornea. Conjunctiva may provide a preferred route for the absorption of large and hydrophilic molecules [14,15]. However, it is highly vascularized, and drugs penetrating the conjunctiva may be absorbed systematically directly from the conjunctival sac or the nasal cavity and reach general blood circulation rather than intraocular segments [8,16]. It may cause significant drug loss into the systemic circulation thereby lowering its ocular bioavailability. To improve the efficacy of drug delivery via the topical route, high drug concentrations and repeated instillations are often required to reach the desired therapeutic effects, which can cause side effects and poor patient compliance [16]. In particular, children have a much higher risk of systemic side effects because their physiological development differs from that of adults, and dosing of ophthalmic drugs is not weight adjusted [17].

The cornea is the most significant mechanical and chemical barrier to ophthalmic drugs. It is an optically transparent avascular structure, 0.5−0.7 mm thick, consisting of five layers: epithelium, Bowman’s membrane, stroma, Descemet’s membrane, and endothelium [18] (Figure 2). The layers forming substantial permeability barriers are epithelium and stroma. The external corneal stratified multilayer epithelium is a hydrophobic layer with tight junctions between epithelial cells which forms a tight barrier for the hydrophilic drugs [19]. The high lipophilicity of the epithelium offers the penetration of up to 90% of lipophilic drug molecules and only about 10% of hydrophilic molecules [20]. The presence of drug-degrading enzymes in the epithelium represents another reason for low topical drug bioavailability [21,22,23]. The next layer placed right underneath the epithelium is the Bowman’s membrane—an acellular layer, consisting primarily of collagen. The middle corneal layer is a stroma forming about 90% of the total thickness of the cornea. It consists of collagen, other proteins, mucopolysaccharides, and nearly 80% of water. As opposed to the epithelium, stroma limits the penetration of hydrophobic drugs. Next to the stroma is the Descemet’s membrane supporting the internal corneal layer—endothelium, a single layer of epithelia-like cells important in regulating stromal hydration, but a weaker barrier for the drugs than the external layers [20].

The principal problems in ocular drug delivery via topical administration are poor permeability of tissues and short residence time in the tear film. So, two main strategies have been followed to improve ocular bioavailability upon topical administration: (a) increasing pre-corneal retention time, and (b) enhancing corneal, scleral, and/or conjunctival drug permeability [2]. To overcome the ocular drug delivery barriers and improve the drug bioavailability, various conventional and novel drug delivery systems have been developed such as emulsions, ointments, suspensions, aqueous gels, nanomicelles, nanoparticles, liposomes, dendrimers, implants, contact lenses, nanosuspensions, microneedles, in situ thermosensitive gels, etc. Among these, nanosize carriers are the most attractive, as they could enhance drug permeability across the blood-aqueous barrier and cornea, prolong drug contact time with ocular tissues, deliver drugs to a specific tissue in a controlled manner, protect drugs from degradation and metabolism to enhance drug stability, sustain drug release for a long time, diminish toxicity and side effects, maintain long shelf-life, while not needing to reconstitute or surgical removal [24].

Drug-loaded nanoparticles/hydrogels do not enter cells via diffusion. The endocytosis pathway is related to the penetration of drug-loaded nanoparticles/hydrogels into the cell, and the relationship between endocytosis and nanoparticle-based drug delivery has been revealed by many researchers. The interactions between the nanoparticles and the cell membrane generate forces of different origins and lead to the membrane wrapping of the nanoparticles followed by cellular uptake [25].

Various approaches have been extensively described in several reviews concerning different aspects of ocular drug delivery (e.g., [2,5,11,12,20,26,27,28,29,30,31]).

Here, we present basic data for the last ten years with particular interest to the ocular drug delivery to the anterior segment of the eye. Eye pathologies involving the anterior segment are widely distributed and include cataracts, dry eye syndrome, inflammatory diseases, infectious diseases, glaucoma, tumors, trauma (including eye burns), congenital and acquired abnormalities, and ocular manifestations of systemic diseases.

## 2. Polymeric Nanoparticles

Over the past decades, polymeric nanoparticles have become widespread in the field of drug delivery [8,13,29,30,32,33,34,35,36]. Polymeric nanoparticles are defined as particles with a diameter of less than 1000 nm, consisting of various biodegradable polymeric materials. Nanoparticles need to be retained in the conjunctival sac, following topical instillation and subsequent release of the drug from the drug delivery system to achieve sustained drug release and long-term therapeutic activity. If the drug is washed out of the nanoparticles too quickly or too slowly, there would be either insufficiently sustained release of the drug or the concentration of the drug in tears might be too low to ensure the adequate healing effect. For effective retention, it is important to obtain nanoparticles from bioadhesive materials, otherwise, drug-containing nanoparticles would be eliminated from the precorneal site almost as quickly as a drug in solution.

As a basis for polymer nanoparticles, the following polymers are usually used: poly(D,L-lactide-co-glycolide) (PLGA); poly(ε-caprolactone) (PCL); polyacrylamide; Eudragit^®^ (RS100 and RL100); polycyanoacrylate (PCA); polymethylmethacrylate; as well as natural polymers, e.g., chitosan, gelatin, sodium alginate, and albumin. Ophthalmic medications can be embedded directly into the nanoparticle matrix or attached to the surface of the nanoparticle.

Drug-loaded polymeric nanoparticles can represent nanospheres, where a drug is evenly distributed over the polymer matrix, or nanocapsules, where a drug is enclosed within the polymer shell. Besides, hydrophilic polymers such as polyethylene glycol (PEG) and polyvinylpyrrolidone (PVP) can bind to mucins by hydrogen bonds and/or electrostatic interactions. This ability could provide the increased contact time of the drug carrier with the ocular surface. A long period of contact is needed for the improved bioavailability of the ophthalmic drug and, in general, for the duration of the drug’s action and controlled release of the drug from the delivery system.

The first generation of polymeric nanoparticles from the acrylic polymer PCA demonstrated sustained release of an included drug, as well as retention of the drug on the corneal surface [37,38]. However, after the discovery of PCA toxicity [39], polyacrylate (second generation of nanoparticles) was considered to be more perspective [40]. Then, it was demonstrated that nanoparticles consisting of polyesters (PCL and PLGA) are well tolerated by the eyes [41], as well as nanoparticles consisting of hydrophilic polysaccharides, such as hyaluronic acid and chitosan [42]. As the interaction of nanoparticles with the cornea is a key for long-term drug action, the third generation of nanoparticles has been developed which represent nanoparticles coated with a polymer having certain functional groups.

Table 1 illustrates some polymeric nanoparticles on the base of both natural and synthetic polymers with included ophthalmic drugs.

### 2.1. Natural Materials

#### 2.1.1. Chitosan

The surface charge of the drug carrier strongly affects the efficiency of the drug penetration into the eye and positively charged carriers (particles) have an advantage over negatively charged or neutral carriers (particles) due to their estimated interaction with mucins and, as a result, a longer retention time of the drug on the surface of the eye [84]. Therefore, cationic carriers, such as mucoadhesive chitosan, appear to be promising for drug delivery to the eye [45,85]. A mucoadhesiveness of chitosan is associated not only with electrostatic interactions but also with hydrogen bonds and hydrophobic effects [86].

Chitosan is a linear polysaccharide composed of D-glucosamine (deacetylated unit) and N-acetyl-D-glucosamine (acetylated unit) which is produced commercially by deacetylation of chitin. On average, the molecular weight of chitosan is 3.8–20 kDa. Chitosan can be applied as chitosan nanoparticles containing ocular drugs [48,49,50,51,52,59] and as a modifier (coating agent) of other nanocarriers, such as alginate [54] and PLGA [87]. Chitosan-based particles are usually obtained by ionotropic gelation with a polyvalent anion, most often sodium tripolyphosphate, as a crosslinking agent [49,59,88]. Chitosan particles are characterized by a positive charge, usually more than +10 mV, and wide size distribution from tens to hundreds nm [48,49,51,52].

During the last decade, chitosan has been widely used to develop nanoparticles for drug delivery to the anterior segment of the eye. It showed promising results in the delivery of a variety of drugs, such as β-blockers timolol [43] and carteolol [44,89], anti-glaucomatous agent, dorzolamide [45], anti-viral drug ganciclovir [46], immunosuppressant cyclosporine A [47], non-steroid anti-inflammatory drug indomethacin [90], etc. Table 1 shows some characteristics of chitosan nanoparticles containing various drugs.

In vitro experiments showed that drug compounds included in chitosan particles were slowly released from the particles in physiologically relevant conditions in a time from half an hour to up to two days [49,51]. A model fluid simulating tear fluid and containing mucin and lysozyme did not affect the stability of chitosan particles [48], but a decrease in the ζ-potential of the particles indicated their interaction with mucin [48,52]. Chitosan particles containing rosmarinic acid, which has anti-inflammatory and anti-viral activity, did not show cytotoxicity towards the cells of the retinal pigment epithelium (ARPE-19) and the human corneal cell line (HCE-T) [52]. At the same time, chitosan particles containing diclofenac, a nonsteroidal anti-inflammatory drug, exhibited high antibacterial activity against *S. aureus* and *B. subtilis* [49]. The strong anti-inflammatory drug, dexamethasone, showed even more pronounced anti-inflammatory properties when incorporated into chitosan particles as it strongly suppressed the release of NO, TNF-α, and interleukin 6 from macrophage cells [51].

The data presented in Table 1 demonstrate that the inclusion of ocular drugs in chitosan particles increased the contact of the formulations with mucins on the ocular surface [48,52], increased the residence time of the drugs in aqueous humor after single instillation [50], and significantly increased the rate of drug penetration through the corneal cells in the ex vivo experiments [59]. Moreover, it was shown that the drugs decreasing intraocular pressure (IOP) performed this function more effectively in the form of the drug-containing chitosan particles [59].

Synthetic polymers are usually not mucoadhesive, so this causes some limitations in bioavailability at the surface of the cornea. To overcome this limitation, several synthetic polymer formulations have been combined with chitosan. For example, chitosan has been used as a coating agent for PLGA nanoparticles containing forskolin; this way, a greater IOP lowering effect and duration of action were achieved [87]. Chitosan-coated PLGA nanoparticles have also been used to deliver glucocorticosteroid, fluocinolone acetonide, to the anterior segment of the Albino rabbit eye [91]. The results showed that the chitosan coating increases the bioavailability of the particles and slows down the drug release from PLGA nanoparticles. Due to its hydrophilic properties, chitosan is not suitable for encapsulating hydrophobic drugs. For this purpose, copolymers of chitosan with grafted PLA and PEG were suggested as ophthalmic drugs carriers to improve drug delivery [92,93].

Chitosan-coated PCL nanoparticles loaded with carbonic anhydrase inhibitor, dorzolamide, were developed for improved ocular delivery. The particle size, polydispersity index, ζ-potential, and encapsulation efficiency of the nanoparticles were found to be 192.38 ± 6.42 nm, 0.18 ± 0.04, +5.21 ± 1.24 mV, and 72.48 ± 5.62%, respectively. The corneal flux experiment showed significant enhancement in permeation across goat cornea. The particles demonstrated 3.7 fold higher mucoadhesive strength compared to the control, were non-irritant and safe for ocular administration [45].

#### 2.1.2. Alginate

Alginate, like chitosan, is a natural linear block polymer composed of blocks of (1→4)-linked β-(1-4)mannunoric acid and β-(1-4)guluronic acid and characterized by a molecular mass from 10 to 600 kDa. Despite its negative charge, alginate possesses good mucoadhesive properties due to its ability to make contact with mucin glycoproteins via H-bonds by a large number of hydroxyl groups [13]. Bioadhesive anionic alginate nanoparticles were prepared for the drug delivery and sustained release of the anti-glaucomatous agent, brimonidine [55]. Nanoparticles were prepared using a spontaneous emulsification solvent diffusion method. The in vivo study revealed that the IOP-lowering effect of the formulation lasted for more than 25 h after a single topical instillation [55].

Alginate, however, has low stability, which limits its use in the design of drug delivery systems for prolonged drug release. For this reason, a mixture of chitosan and alginate was suggested for nanoparticle formulations. For example, alginate-chitosan microspheres were shown to increase the retention time of azelastine, a drug used for the treatment of allergic conjunctivitis, in the conjunctival sac and on the corneal surface, as well as to improve therapeutic efficacy in vivo [53]. The microspheres were characterized by an average particle size from 3.55 to 6.70 µm and ζ-potential from +24.55 to +49.56 mV. These alginate-chitosan microspheres showed 73% drug entrapment and 65% mucin binding efficiency and revealed a controlled drug release over 8 h. The advantages of the complex chitosan-alginate nanoparticles were also demonstrated in [56], where the particles containing glucocorticosteroid, betamethasone, were obtained. The mean particle size ranged from 16.8 to 692 nm and ζ-potential generally ranged from +18.49 to +29.83 mV depending on the synthesis conditions. The in vivo studies carried out for two selected formulations showed the release of 84% and 59.5% of the drug over 12 h [56].

Another study [54] compared the effect of the additional chitosan coating of chitosan-alginate nanoparticles on the delivery of 5-fluorouracil (5-FU), cytotoxic medication. Unmodified chitosan-alginate nanoparticles did interact with mucin while an enhanced viscosity was observed after coating of nanoparticles with chitosan. Chitosan-coated nanoparticles provided a sustained release of 5-FU after the high burst effect. The experiments in vivo showed a significantly higher level of 5-FU in aqueous humor of rabbits as a result of drug delivery by chitosan-coated chitosan-alginate nanoparticles. The enhanced mucoadhesiveness of chitosan coating of chitosan-alginate nanoparticles results in higher bioavailability as compared to the uncoated nanoparticles [54].

#### 2.1.3. Other Natural Polymers

Other the most common natural polymers used as drug carriers are albumin, gelatin, and hyaluronic acid. Albumin particles containing anti-inflammatory piroxicam caused a 1.8-fold increase in bioavailability after its topical instillation in the eye of rabbits in comparison with the standard commercial eyedrops [94]. Albumin has also been combined with chitosan to create microspheres loaded with anesthetics tetracaine [95] and atropine [58]. It appeared that microencapsulated tetracaine and atropine significantly increased the duration and peak value of the effect of drugs compared to standard drug solutions.

Gelatin nanoparticles were also selected for topical drug delivery to the eye because of their biocompatibility and biodegradability. It was shown that positively charged gelatin nanoparticles with a size of 180.6 ± 45.7 nm were efficiently adsorbed on the negatively charged cornea without irritating the eyes of the rabbits and can be retained there for a long time [60]. Development of gelatin nanoparticles loaded with moxifloxacin resulted in the generation of positively charged nanoparticles with ζ-potential +24 ± 0.12 mV and a narrow particle size of 175 ± 1.11 nm [61]. In vitro drug release from these nanoparticles exhibited an initial burst in the first hour followed by a slow controlled release of the drug for the subsequent 12 h. The in vivo tolerance tests revealed that the drug-loaded nano-formulations were non-irritant to the ocular tissues indicating their safety. Anti-bacterial activity of the suspension of nanoparticles with moxifloxacin was more effective against *S. aureus* than the MoxiGram, a commercially market product [61]. Cationic gelatin can be also combined with polyanions, chondroitin sulfate, or dextran sulfate, to obtain hybrid nanoparticles. This approach was applied to transfect ocular epithelial cells by the nanoparticles using a plasmid specially designed to encode human MUC5AC mucin [62]. MUC5AC mRNA and protein were detected in conjunctival cells after in vitro transfection of the nanoparticles. Administration of the nanoparticles in the experiments in vivo resulted in significantly higher MUC5AC expression in the conjunctiva compared to untreated control and naked plasmid [62].

### 2.2. Synthetic Materials

#### 2.2.1. Eudragit

Eudragit is the trade name used for synthetic copolymers derived from esters of acrylic and methacrylic acid. Eudragit polymers present great versatility according to the functional groups in the side chain of the polymer. These polymers have been used to successfully deliver the drugs to the ocular surface, e.g., anti-inflammatory aceclofenac [63,64] and diclofenac [96], as well as anti-glaucomatous acetazolamide [65]. Eudragit RL 100-based and Eudragit RS 100-based nanoparticles containing aceclofenac were obtained by nanoprecipitation [63,64]. The studies in vivo involving arachidonic acid-induced ocular inflammation in rabbits revealed significantly higher inhibition of polymorphonuclear leukocytes migration and lid closure scores by the nanoparticle formulations compared with the aqueous solution of aceclofenac. The formulations were quite stable to ensure two-year shelf life at room temperature.

Acetazolamide-loaded Eudragit^®^ RL 100 nanoparticle suspension has a more substantial and prolonged decreasing effect on the IOP than acetazolamide in solution [65]. The short-term study showed that the formulations did not change their physicochemical properties after 6 months of storage at various temperatures. Eudragit RL 100-based nanoparticles loaded with immunosuppressor, tacrolimus, were also obtained for topical ocular application [66]. The nanoparticles exhibited monomodal size distribution with a mean diameter of 104 ± 1 nm, positive ζ-potential, and mucoadhesive properties. The in vivo safety investigation showed no signs of ocular irritation both by ophthalmological examination and histopathological study. The authors declared the slow release of tacrolimus from the particles and much better penetration of tacrolimus-loaded nanoparticles to the eye than the drug in solution [66].

#### 2.2.2. PLGA

PLGA is a synthetic copolymer of poly(lactic acid) and poly(glycolic acid) known for its biodegradability and biocompatibility. PLGA nanoparticles were developed for the delivery of glucocorticoid fluorometholone [67] and anti-inflammatory aceclofenac [68] to the eye. Fluorometholone-loaded PLGA nanoparticles were characterized by a negative ζ-potential, about −30 mV, and an average size below 200 nm. These nanoparticles had significantly greater anti-inflammatory effects than the commercial formulation, Isoptoflucon^®^ and nanoparticles increased drug penetration toward the vitreous [67]. PLGA-nanoparticles loaded with aceclofenac and obtained by direct precipitation method were characterized by the particle size from 162.6 to 244.13 nm and by negative ζ-potential of −21.5 to −25.5 mV. An in vivo ocular anti-inflammatory study in the rabbit eyes confirmed better efficacy of drug-loaded nanoparticles compared with the drug solution [68]. PLGA nanoparticles loaded with tacrolimus were also designed for topical ocular instillations by two research teams [69,70]. Alshamsan et al. developed PLGA-tacrolimus nanoparticles by the emulsification-diffusion method. It was shown that the entrapment of tacrolimus by nanoparticles significantly enhanced its ocular bioavailability [69]. Benita et al. developed PLGA-tacrolimus nanoparticles by a well-established solvent displacement method. It is worth noting that multiple-dose ocular instillation of the nanoparticles in rat eyes allowed high tacrolimus levels within the eye with very low concentrations of tacrolimus in plasma [70].

PLGA nanoparticles loaded by anti-inflammatory drug, pranoprofen, were characterized by a mean particle size of 350 nm, with a polydispersity index below 0.1, and a negative charge of −7.41 mV. The putative irritancy of nanoparticles was examined on New Zealand white rabbits which tolerated well the topical instillations of nanoparticles suspension to the eye; moreover, nanoparticles significantly reduced the ocular edema [71]. PLGA nanoparticles encapsulating flavonoid, xanthohumol, were found to be cytoprotective against oxidative stress in vitro, and significantly reduced ocular surface damage and oxidative stress-associated DNA damage in corneal epithelial cells in the mouse desiccating stress/scopolamine model for dry eye disease in vivo [72]. Besides, the antioxidant compound, ferulic acid, was also loaded into PLGA nanoparticles because of its poor solubility and, therefore, low therapeutic efficacy. The authors demonstrated the absence of cytotoxicity of the obtained stable formulation on retinal pericytes and epithelial cells and suggested that ferulic acid within PLGA nanoparticles could be used in ophthalmology [97].

Thus, in general, PLGA nanoparticles increase drug bioavailability, but PLGA copolymer is not mucoadhesive. Therefore, mucoadhesive polymers, e.g., chitosan or PEG, can be used as PLGA modifiers to enhance the affinity of the polymer to mucus [98,99]. So, chitosan-coated PLGA nano- and microparticles were obtained by the double emulsion solvent evaporation method [57] and by capillary microfluidic techniques [98]. The chitosan-coated PLGA microparticles showed strong instant adhesion to mucins [98], while chitosan-coated PLGA nanoparticles containing bevacizumab, a drug against a vascular endothelium growth factor, demonstrated significantly higher flux through goat sclera [99]. Microparticles composed from PLGA and PEG and containing anti-glaucomatous dorzolamide showed a 35% greater IOP decrease and >2-fold prolongation of this effect after topical instillation of particles suspension to the eye, as compared to Trusopt^®^, the commercial eye drops of dorzolamide [73].

As mentioned above, in the chapter on chitosan, to increase the mucoadhesiveness of fluocinolone acetonide-loaded PLGA nanoparticles, surface modification was done using not only chitosan but also stearylamine. Pharmacokinetic studies on Albino rabbits’ eyes using HPLC indicated that the prepared novel chitosan-coated PLGA nanoparticles showed rapid and extended drug delivery to the eye in comparison with unmodified PLGA nanoparticles [91].

#### 2.2.3. PLA

Poly(lactide) (PLA) is a synthetic hydrophobic polyester synthesized by ring-opening polymerization of lactide. PLA drug delivery systems have been widely used for various biomedical applications [100]. Moreover, because of PLA hydrophobic nature poorly water-soluble drugs are encapsulated in the particles formed by PLA better than hydrophilic ones. However, biodegradation of PLA is relatively low in comparison with other polymers [101], this is why PLA is usually grafted with other polymers to tailor its biodegradability [56].

Thus, the surface of the hybrid PLA-dextran nanoparticles was functionalized with a mucoadhesive ligand, phenylboronic acid. These particles were characterized by particle size ranging from 25 to 28 nm. Using cyclosporin A as a model drug, it was shown that nanoparticles encapsulated up to 13.7 wt% of the drug and exhibited sustained release for up to 5 d in vitro at a clinically relevant dose [102].

## 3. Polymeric Nanomicelles

Nanomicelles are composed of amphiphilic molecules (surfactants or block copolymers) that self-organize in an aqueous solution to form organized supramolecular structures [103,104,105]. During micellization, hydrophobic segments join to form a core region, while hydrophilic segments form a hydrophilic shell of micelles. Micelles of various sizes (10–1000 nm) and shapes can be obtained depending on the molecular weight of the polymer forming the core and crown-forming blocks. Micelle structure can be adapted to obtain unique properties considering delivery requirements, e.g., prolonged stability of micelles in tear fluid to increase the contact time with the cornea. A small particle size, ease of preparation, a high ability to encapsulate a drug made nanomicellar technology popular for ocular drug delivery [106,107]. Recently, nanomicellar approaches have been used with both polymer and surfactant to deliver locally small molecules as well as genes to the anterior segment of the eye [103,108].

In particular, most of the polymeric micelles used in drug delivery consist of amphiphilic di-block (hydrophilic-hydrophobic) polymers, tri-block (hydrophilic-hydrophobic-hydrophilic) polymers, graft (hydrophilic-hydrophobic), and ionic (hydrophilic-ionic) copolymers. For the majority of these systems, PEG is the primary hydrophilic segment. Also, the hydrophilic outer part of polymeric micelles can be composed of poly(ethylene oxide) (PEO), poly (acryloylmorpholine), poly(trimethylene carbonate), or poly(vinylpyrrolidone). Block copolymers such as PEO-poly(L-amino acids) provide an additional advantage to modify the properties of core-forming blocks. For example, the functional groups of the block are capable of chemical conjugation with a drug allowing efficient delivery of therapeutic doses. The hydrophobic core of polymeric micelles is usually made up of Pluronics (poly(ethylene oxide)-b-poly(propylene oxide)-b-poly(ethylene oxide)), poly(L-amino acids), such as poly(L-aspartate) and poly(L-glutamate), polyesters, such as poly(glycolic acid, PLA, PLGA, and PCL.

One example of potent ophthalmic formulations on the base of nanomicelles is nanomicelles composed of PLA grafted with poly(methacrylic acid) and phenylboronic acid for the delivery of cyclosporin A [109]. The nanomicelles showed low cytotoxicity in vitro against human corneal epithelial cells and negligible ocular irritation in Sprague-Dawley rats, suggesting good biocompatibility with the corneal surface. Thus, these micelles show the potential to significantly improve the bioavailability of topically applied ophthalmic drugs [109].

Anti-inflammatory drug pimecrolimus, which selectively inhibits the production and release of cytokines and mediators from T-lymphocytes and mast cells, was loaded into nanomicelles based on PEG-PCL [76]. The micelles had a mean particle size of 37.85 nm, which perfectly meets the requirement for corneal permeation. The encapsulation efficiency of pimecrolimus was 97.9% ± 1.26%. Pimecrolimus-loaded nanomicelles demonstrated sustained drug release and were very effective in the treatment of keratoconjunctivitis on a mouse model [76].

Putative synergistic performance of mixed polymeric micelles made of linear and branched poly(ethylene oxide)-poly(propylene oxide) for more effective encapsulation of anti-inflammatory agent, lornoxicam, was studied in [83]. The use of micelles allowed to increase the solubility of this hydrophobic drug by about 73-fold. Further investigations by histopathological and confocal laser studies revealed the non-irritant nature and good corneal penetrating of the proposed nano-formulation [83].

In another study, myricetin, a flavonoid with antioxidant properties, was encapsulated into polyvinyl caprolactam-polyvinyl acetate-polyethylene glycol graft copolymer (PVCL-PVA-PEG) micelles to increase its solubility, aqueous stability, and bioavailability. PVCL-PVA-PEG micelles significantly enhanced both solubility and stability of myricetin. Myricetin micelles showed quite low toxicity to the human corneal epithelial cells. Remarkable improvements in cellular uptake and antioxidant activity in vitro, as well as in vivo improvement of permeation into rabbit eye cornea were also observed [78].

For the treatment of dry eye syndrome, one of the most common eye diseases, methoxy-PEG-PLA micelles as alternative vehicles for cyclosporin A solubilization and its delivery to the eye were suggested. The in vitro experiments showed that lyophilized cyclosporin A-loaded micelles were stable for at least 3 months and the release profile showed a sustained release manner of cyclosporin A. In vivo ocular distribution studies demonstrated that the micellar formulations exhibited a 4.5-fold increase in retention effect in eyes compared with 0.05% cyclosporin A emulsion [77].

As a novel ophthalmic formulation anti-angiogenic drug, axitinib, which represents a class of tyrosine kinase inhibitors, was loaded via the amphiphilic copolymer PEG-PCL, improving its solubility in water. Axitinib-loaded micelles showed low toxicity in concentration gradient assays. Anti-angiogenic efficacy of axitinib-loaded micelles was tested on corneal neovascularization after alkali burn of rat eyes, with dexamethasone as a positive control. It was shown that axitinib-loaded micelles had anti-angiogenic effects without obvious tissue toxicity. The authors suggested that, as a class of tyrosine kinase inhibitors, axitinib-loaded micelles can be used in the treatment of ocular neovascular diseases [79].

For the treatment of eye inflammatory disease, uveitis, dexamethasone-loaded PCL-PEG-PCL micelles were developed by film hydration method. Cellular uptake was studied using flow cytometry and fluorescence microscopy. After a lag-time, the dexamethasone-loaded micelles reduced the clinical symptoms of uveitis, however, the differences with commercial eye drops did not reach statistically significant levels [80].

Polymer nanomicelles could also serve as carriers of high molecular weight compounds, such as enzymes. These formulations designed for the drug delivery to the eye were described in [81,82]. Thus, the so-called “nanozyme” formed by self-assembly of the antioxidant enzyme, superoxide dismutase 1 (SOD1) with PEG-poly-L-lysine_50_ block copolymer and further stabilized by cross-linking was used as a carrier for the delivery of SOD1 into ocular tissues for the treatment of inflammation processes in the eye. The SOD1-nanozyme appeared to be much more effective compared with the free enzyme in decreasing uveitis manifestations in rabbits, such as the intensity of corneal and iris edema, hyperemia of the conjunctiva, lens opacity, the amount of fibrin clots, and the protein content in aqueous humor. Topical instillations of SOD1-nanozyme significantly decreased inflammation both in the outer and inner parts of the eye as determined using scores of the clinical manifestations of uveitis, multiple biochemical parameters, and histological analysis [81].

Recently, double-coated SOD1-containing nanomicelles based on multilayer polyion complexes and called nanoSOD1 were synthesized [82], These cross-linked nanomicelles were characterized by high storage stability and pronounced therapeutic effect on immunogenic uveitis in rabbits without side reactions such as eye irritation; acute, chronic, and reproductive toxicity; allergenicity; immunogenicity, and mutagenicity even in high doses. It was shown during preclinical studies that topical instillations of nanoSOD1 were much more effective compared to the free enzyme in decreasing uveitis manifestations, such as corneal and conjunctival edema, iris hyperemia, and the amount of fibrin clots in the anterior chamber. Moreover, it was shown that nanoSOD1 penetrated anterior eye structures more effectively than SOD1 itself and retained enzyme activity in the eye for a much longer period, decreasing inflammation and restoring antioxidant activity in the eye [82].

## 4. Inorganic Particles

Inorganic nanoparticles suitable for nanomedicine include gold [110,111,112,113,114] and silver [115,116] nanoparticles, cerium dioxide [117,118], silica [119,120], as well as inorganic salts [121,122,123].

Some inorganic nanoparticles can be considered drugs themselves, as well as they can serve as carriers of medicinal compounds. Most often, the proposed particles consist of two regions: a core containing an inorganic component (for example, gold or silica) and a polymer cover for increased bioavailability [111,112,120,124].

### 4.1. Gold Nanoparticles

The advantages of gold nanoparticles are in their chemical stability, the possibility of surface modification, and biocompatibility [110,125]. Biocompatibility and the ability of the cells to internalize various morphologies of gold nanoparticles were assessed with the cells of the retinal pigment epithelium. Nanoparticles in the form of spheres and cubes 50 and 100 nm in size (but not rods) did not show cytotoxicity [126].

It was shown that gold nanoparticles themselves had a positive effect on endotoxin-induced uveitis in rats [114]. Liposomes containing gold nanoparticles with flucytosine were suggested for the treatment of fungal eye infections [113]. For the prevention of cataracts development, gold nanoparticles with N-acetylcarnosine stabilized with polyphenols were obtained [127]. Gold nanoparticles with anti-glaucoma drug, timolol, were included in the contact lens [112]. Also, gold nanoparticles with amfenac stabilized by poly(catechin) were proposed for the treatment of dry eye syndrome [128].

### 4.2. Silver Nanoparticles

Silver nanoparticles are known for their antimicrobial properties [115,129,130], as well as their anti-angiogenic effect due to inhibition of the hypoxia factor HIF-1α in cells, which, in turn, leads to the suppression of the vascular endothelial growth factor VEGF-A function [131]. It has been shown that a suspension of silver particles 15–50 nm in size has a powerful antioxidant effect, which could be potentially used to prevent the development of cataracts [116]. However, it has been shown that silver nanoparticles are rather toxic [132,133]. The toxicity can be associated with the release of silver ions from the surface of the nanoparticles. In particular, it has been shown that silver nanoparticles, when injected into the eye, might inhibit the development of lenses in zebrafish embryos [132], and also cause edema and erythema of the conjunctiva of rabbits [133].

### 4.3. Cerium Particles (CeO_2_)

Nanoparticles based on cerium oxide are practically non-toxic and have a powerful antioxidant effect [118,134]. Cerium oxide nanoparticles have been shown to mimic oxidoreductase enzymes by catalyzing the decomposition of organic substrates and reactive oxygen species. Moreover, the covering of 7.8 nm cerium oxide cores with two poly(sodium acrylate) and four poly(ethylene glycol) (PEG)-grafted copolymers with different terminal or anchoring end groups did not affect the superoxide dismutase-like activity but surprisingly improved peroxidase-like catalytic activities of cerium oxide nanoparticles [135]. It has been shown that cerium nanoparticles penetrate well into the tissues of the eye; in particular, they remain in the retina for up to 1 year after a single intravitreal injection, without causing inflammatory processes [134,136].

The antioxidant effect of cerium oxide in water-soluble cerium oxide-loaded glycol chitosan nanoparticles was assessed in mice primary corneal and conjunctival cells in vitro and in a dry eye murine model in vivo. These particles had no cytotoxic effect and showed improvements on dry eye disease models by stabilizing the tear film, scavenging reactive oxygen species (ROS), up-regulating SOD, promoting and maintaining corneal and conjunctival cell growth and integrity [137]. For the treatment of glaucoma, a formulation was proposed based on the combination of the antioxidant action of cerium particles with the hypotensive action of pilocarpine [138]. Specifically, chitosan and ZM241385 (nonxanthine adenosine receptor antagonist that can selectively bind to A2AR subtype in the ciliary body) were functionalized onto surfaces of hollow ceria nanoparticles, thereby endowing the nanocarriers with a strong capability to open corneal epithelial tight junctions and deliver drug molecules to the ciliary body. These formulations were demonstrated in vitro and in vivo to possess potent antioxidant and anti-inflammatory properties and effectively mitigated the disease progression in experimentally glaucomatous eyes after a single topical application. The effect of a drop on IOP persisted for up to 7 days [138].

### 4.4. Silica Nanoparticles

Silica nanoparticles are among the most studied inorganic nanoparticles for drug delivery. They have a size of 30–300 nm, a large specific surface area, and are chemically stable. Moreover, silica-based particles are biodegradable and can be eliminated from the body after a few days [139].

It was shown that 50–150 nm silica particles did not cause significant cytotoxicity towards corneal endothelial cells at concentrations up to 100 μg/mL [140], however, caused a slight increase in the generation of reactive oxygen species in the cells [141]. When studying the effect of silica particles on trabecular meshwork cells, they were found to be localized in the cytoplasm, without penetration into the nucleus and mitochondrial damage. The particles induced a moderate dose-dependent increase in the activity of lactate dehydrogenase [142]. Intravitreal injection of silica particles effectively reduced abnormal retinal angiogenesis in mice with oxygen-induced retinopathy [143,144].

Modified silica nanoparticles have been used to include anti-glaucomatous drugs. Thereto amino-functionalized mesoporous silica particles containing brimonidine [120] and gelatin-covered mesoporous silica nanoparticles containing pilocarpine [145] were obtained. The resulting particles were shown to slowly release brimonidine in a sustained manner over 8 h. It is important that the particles were mucoadhesive, thus allowing the drug to reside in the tear film for up to 12 h because it adhered to the mucous layer. A decrease in IOP was observed even after 12 h [120]. Gelatin-covered silica nanoparticles with pilocarpine were intracamerally administrated to the glaucomatous eyes. The in vivo studies showed the maintenance of normal IOP in the eyes with ocular hypertension for 21 days [145].

### 4.5. Particles Formed by Inorganic Salts

Promising carriers for drug delivery are calcium carbonate and calcium phosphate [121,122,139,146,147,148,149,150]. This is due to the biocompatibility and biodegradability of these salts [148,151], as well as the simplicity and low cost of the synthesis [123,152].

Calcium carbonate is naturally present in the forms of calcite, aragonite, and vaterite. Among the polymorphic modifications, metastable vaterite is the most important in practice, since it has a high porosity, large surface area, and can rapidly dissolve under relatively mild conditions [123,153,154,155].

Calcium carbonate particles could be obtained by simple mixing solutions containing calcium cations and carbonate anions. By varying the synthesis conditions, such as pH, temperature, and the presence of extraneous reagents, particles of different sizes and morphologies can be obtained [153,156]. For example, when polyols or surfactants are added, exclusively vaterite particles were formed with a size of 1–2 µm [156]. The inclusion of drugs in calcium carbonate particles can be carried out either by co-precipitation during the synthesis of the particles or by adsorption of the drug on the particles [123,139,157]. It is worth noting that calcium carbonate particles possess mucoadhesive properties, which can improve their interaction with the ocular surface [154]. However, the main disadvantage of vaterite particles is their tendency to quickly recrystallize into non-porous calcite crystals [123,158], which is accompanied by a significant decrease in the surface area of the particles [155] and a release of the included drug [123,155]. Nevertheless, vaterite particles loaded by SOD1 were suggested in [123] for possible further treatment of eye inflammation. The enzyme was released from the particles in two phases: in the first 1.5 h, about 80% of the drug was released. After 24 h, vaterite recrystallized into non-porous calcite, this process was accompanied by the release of remaining SOD1 into solution [123].

Nanoparticles of calcium phosphate are of particular interest as they are highly biocompatible [159]. Calcium phosphate is a structural material of the bone tissue of the human body. Dissolution of calcium phosphate carriers in slightly acidic media can induce intracellular drug release, while the resulting carrier components Ca^2+^ and PO_4_^3−^ are non-toxic and do not accumulate in the tissues [160].

There are different methods of obtaining calcium-phosphate particles with various morphology and sizes: precipitation from the solutions of water-soluble salts containing phosphate anions and calcium cations [121,150,161,162,163]; hydrothermal method using microwave radiation [164]; microemulsion method in the water/hexane/polyoxyethylene-(5)-nonylphenyl ether system [165,166,167], enzymatic hydrolysis of calcium glycerophosphate by alkaline phosphatase [168]. The simplest method is just mixing the solutions of salts containing phosphate and calcium ions with further ultrasound treatment [121,150,161,162,163].

Several studies have considered the possibility of using calcium phosphate nanoparticles in ophthalmology. Thus, methazolamide, an inhibitor of carbonic anhydrase, was included in calcium phosphate nanoparticles, which were characterized by a diameter of 256 nm and a ζ-potential of −30 mV. In vivo experiments on rabbits demonstrated that methazolamide in the composition of particles decreased IOP much more effectively compared with an aqueous form [146].

Calcium phosphate nanoparticles were shown to be able to contain both low molecular weight and high molecular weight substances. Thus, such particles containing 316 Da β-blocker timolol [121,161], 406 Da inhibitor of angiotensin-converting enzyme, lisinopril [121,122], which is also able to decrease IOP at topical instillations [169], and 32 kDa superoxide dismutase 1 [150] were reported. The particles were further coated with a disaccharide, cellobiose [122,161], or with a polysaccharide, 89 kDa chitosan [121]. The particles containing different compounds were characterized by a size of 140–300 nm [121,122,150,161]. Uncoated calcium phosphate nanoparticles with encapsulated SOD1 had a negative ζ-potential of about −4 mV. Calcium phosphate nanoparticles containing lisinopril and timolol and coated with cellobiose had a negative ζ-potential from −7 to −17 mV [122,161], while the same particles coated with chitosan had a positive ζ-potential +16 mV [121].

The efficiency of lisinopril incorporation (by co-precipitation during the synthesis of the particles) into calcium phosphate nanoparticles coated with chitosan or cellobiose was much higher than that of timolol (also incorporated into the particles by co-precipitation) indicating specific interactions of lisinopril with inorganic particles [121,122,161]. At the same time, the percentage of lisinopril included in chitosan-coated calcium phosphate particles was higher than in cellobiose-coated particles, apparently due to electrostatic interaction between chitosan “coat” and lisinopril [121].

Experiments in vivo have demonstrated that the inclusion of timolol and lisinopril into calcium phosphate nanoparticles led to a stronger drop of IOP in rabbits, as well as to prolongation of the drug action compared to that caused by soluble forms of the drugs [121,122,161].

Also, the experiments in vivo demonstrated a stronger anti-inflammatory effect of SOD1 included in calcium phosphate nanoparticles compared to native SOD1 in experimental immunogenic uveitis in rabbits [150]. Namely, iris and cornea edema, edema and hyperemia of the eyelids, the amount of fibrin in the anterior chamber of the eye, and the presence of posterior synechiae decreased remarkably [150].

## 5. In Situ Hydrogels as an Additional Carrier of Drug-Loaded Nanoparticles

The promising way to solve the problems of the rapid and extensive precorneal loss of the drug instilled in the eye caused by the drainage and the high tear fluid turnover, and to increase the contact time between the drug and the corneal surface is the use of stimuli-responsive hydrogels. They undergo sol-gel conversion «swell» in the presence of a specific biological stimulus (changes in temperature, pH, or electrolyte interaction) and can turn back into solution again when the trigger is removed. Thus, the most efficient drug delivery can be expected if drug-loaded nano-carriers are incorporated into the in situ gel. The sensitivity against the biological stimulus can be enhanced by using a combination of polymers that respond to multiple biological stimuli. Thus, the suspension of nanocarriers in the in situ gel can provide the advantages of both nano- and gel technologies [170]. While the design of such formulations is one of the most challenging paths in the creation of new forms of drugs for ophthalmic delivery, these complex formulations might noticeably extend ocular residence time and improve the bioavailability of ophthalmic medications [171,172,173].

Thermosensitive gels are the most studied of stimuli-sensitive polymer systems in drug delivery. The temperature of sol-gel transition is called the lower critical solution temperature (LCST). Below LCST, the system remains as a solution and above that temperature, the formulation transforms to a gel [172,174,175,176]. So, the putative ophthalmic formulation should be a solution or, more correctly, colloid solution of nanoparticles or nanomicelles at room temperature. After instillation and contact with the eye surface, however, this formulation is expected to form gel containing nanoparticles/nanomicelles with a loaded ophthalmic agent.

Poloxamers or Pluronics^©^—nonionic triblock copolymers composed of a central hydrophobic chain of poly(propylene oxide) flanked by two hydrophilic chains of poly(ethylene oxide)—represent one class of thermosensitive gels. These polymers and their combinations with other polymers have been extensively investigated for ocular drug delivery [177,178,179,180]. Thus, the combination of cationic nanostructured lipid carriers and thermosensitive Poloxamer 188 gel allowed to increase the bioavailability of curcumin by improving corneal permeation and retention capacity from 1.5 up to 5 h in the aqueous humor of rabbits [181].

The combination of nanostructured lipid carriers and a thermoresponsive Pluronic F127 gel was proposed for the ophthalmic administration of an anti-inflammatory drug, ibuprofen. The formulation demonstrated a prolonged in vitro release profile of ibuprofen, good stability, and safety for ocular delivery [182].

Niosomes loaded with the antifungal drug, voriconazole, were incorporated into in situ gelling mixture of span 40 and span 60 with Pluronic F127 and Pluronic L64. The formulations were designed to be placed into the conjunctival sac. They showed prolonged drug release with superior mucoadhesion [183].

Another antifungal medication, natamycin, was loaded into niosomes and then incorporated into thermosensitive in situ gel using Poloxamer 407 and Hydroxy propyl methyl cellulose K4M, which helped to increase precorneal retention time in ex vivo goat’s cornea. The formulation exhibited greater transcorneal permeation and displayed extended drug release up to 24 h [184].

Ethyl cellulose microsponges loaded with an anti-glaucoma drug, acetazolamide, were incorporated into 25% Pluronic F-127 in situ gel for ophthalmic delivery. The formulation could significantly decrease the IOP in rabbit eyes causing no irritation [185].

Another group of the in situ gels are polymer systems the state of which, solution or gel, is controlled by pH-values of the media. Thus, it is expected that the system stays as a solution at lower pH, but undergoes gel formation at pH 7.4, that is the pH value of the tear fluid. However, as eyes become irritant at low pH, there are some limitations for those in situ gels. Nevertheless, several ocular formulations have been developed with the use of pH-sensitive in situ gels.

Norfloxacin-loaded nanoparticulate was presented as in situ gel based on chitosan/carbopol934; the formulation transformed to the gel at physiological pH and exhibited superior performance over the marketed eye drops [171].

Anti-bacterial agent, sparfloxacin, was incorporated into poly(lactic-co-glycolic acid) nanoparticles and, then, into chitosan in situ gel. The formulation showed gelation at pH near 7.2, demonstrated good retention over the entire precorneal area, and cleared at a very slow rate [186].

Gupta et al. designed levofloxacin non-mucoadhesive nanoparticles laden in mucoadhesive chitosan in situ gel. The preparation formed a gel at pH 7.15, showed an improved retention time compared with separate levofloxacin, levofloxacin in the in situ gel, and levofloxacin in nanoparticles without gel [187].

Chitosan in combination with polyol-phosphates has also been reported to show temperature-sensitive in situ gelling properties. The transition of this mixture into the gel was no longer influenced by the pH of the medium, it remained in a liquid state at low temperatures and underwent sol-gel transition at body temperature [188,189].

For instance, chitosan-based nanoparticles medicated with 5-fluorouracil were introduced into thermosensitive ophthalmic chitosan/β-glycerophosphate hydrogel also medicated with 5-fluorouracil. The formulation provided a sustained release of the drug and increased bioavailability in the in vivo experiments [190].

A temperature-sensitive gel composed of chitosan, carboxymethyl chitosan, and glycerophosphate loaded with chitosan microspheres encapsulating levofloxacin was safe and provided a good bioavailability of the drug [191].

Another thermosensitive formulation was obtained by incorporating the nanostructured lipid carriers into hydroxypropyltrimethyl ammonium chloride chitosan/β-glycerophosphate hydrogel. This composition was suggested for dexamethasone ocular delivery and showed a rapid solution-to-gel transition at 35 °C. Nanocarriers and stimuli-responsive gel coupled their properties to produce an interesting sustained-release formulation for ophthalmic application [192].

One more example of the combination of drug-loaded nanoparticles and in situ gels is the development of poly(lactide-co-glycolide) nanoparticles loaded with ketoconazole with subsequent incorporation of the particles into chitosan/alginate in situ gel. Gelation occurred under the influence of ionic, thermosensitive, or pH interaction. This combination displayed a sustained and greater drug release compared to free drug formulations and improved antifungal activity in comparison to pure drug formulations [193].

## 6. Potential Ocular Nanomedicine

**Figure 3:**
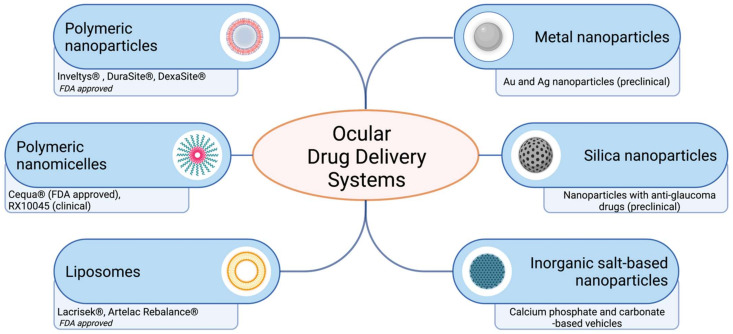
Schematic representation of ocular drug delivery systems for topical administration. Recently FDA approved and developed ocular drug systems presented. Created with BioRender.com.

Over the past decade, ocular drug delivery systems for topical instillation have been extensively investigated. Many nanoformulations for the treatment of eyes have been developed and commercialized in the market (Figure 3, Table 2). Some nanoformulations, such as drug-loading emulsions, are still slowly developing for treatment, even though drug-free nanoemulsion has been approved. The current ocular nanoformulations marketed products are Restasis^®^ (nanoemulsion of cyclosporine A), Cyclokat^®^ (cationic nanoemulsion of cyclosporine A), Cequa^®^ (cyclosporine A ophthalmic nano micellar solution), DUREZOL^®^ (difluprednate nanoemulsion), SYSTANE^®^ (Propylene glycol-based nanoemulsion,) Lacrisek^®^ (vitamin A and vitamin E liposomal spray), Artelac Rebalance^®^ (vitamin B12 liposomal eye drops).

One of the important examples of FDA-approved drugs that have entered the market over the past decade is Inveltys^®^ (KPI-121 1.0%, Kala Pharmaceuticals). Inveltys^®^ is a nanosuspension of loteprednol etabonate (LE) delivered by a proprietary nanoparticle-based formulation referred to as mucus-penetrating particles. A preclinical study of the pharmacokinetics of LE-loaded nanoparticles showed a three-fold increase in C_max_ in the cornea, iris/ciliary body, aqueous humor, and retina compared to LE [194]. In two randomized controlled clinical trials (and NCT02793817), 386 subjects were treated with KPI-121 1% and 325 were treated with placebo following cataract surgery. Combined data analysis showed that significantly more participants treated with KPI-121 vs. vehicle achieved complete resolution of anterior chamber cells at days 8 and 15 and complete clearing of ocular pain at days 4, 8, and 15. KPI-121 1% ophthalmic suspension was effective in resolving postoperative ocular inflammation and pain in patients following cataract surgery [195].

DuraSite technology (Sun Pharma, Alameda, CA, USA) represents a mucoadhesive ocular drug delivery system consisting of a synthetic polymer of cross-linked polyacrylic acid and polycarbophil. Both clinical and nonclinical studies have shown the DuraSite drug delivery system to be safe and non-toxic. The DuraSite technology was used to deliver Bromfenac, a potent topical nonsteroidal anti-inflammatory drug (BromSite), for the treatment of postoperative inflammation and ocular pain. Bromfenac 0.075% in DuraSite was safe, well-tolerated, and effective at reducing inflammation and preventing pain associated with cataract surgery [196]. DexaSite is a nanoformulation of dexamethasone in DuraSite 2, which uses the same polycarbophil polymer in DuraSite with the addition of chitosan, to achieve greater viscosity in comparison with DuraSite [197].

OCS-01 (Dexamethasone Cylcodextrin Nanoparticle Ophthalmic Suspension) is a nanoformulation of dexamethasone with cyclodextrin designed to treat inflammation and pain following cataract surgery. Cyclodextrins form complexes with the lipophilic dexamethasone, increasing the solubility of dexamethasone. In a recent randomized, vehicle-controlled Phase II trial (NCT04130802) in 153 patients following cataract surgery, OCS-01 applied once a day achieved a higher percentage of eyes with the absence of anterior chamber inflammation and a higher percentage of eyes with no pain compared to vehicle at day 15.

RX-10045 nanomicellar solution (Auven Therapeutics) is an aqueous micellar dispersion of an isopropyl ester prodrug of resolvin E1. A Phase II randomized clinical trial was performed (NCT02329743) to assess the safety and efficacy of RX-10045 (0.05% and 0.1%) compared to placebo for the treatment of post-cataract surgery pain and ocular inflammation. Both formulations of RX-10045 were not significantly better than the placebo group in achieving the primary endpoint of clearing anterior inflammation at day 8 post-cataract surgery (22.8% in both treatment groups compared to 16.7% in the placebo group).

## 7. Conclusions

**Figure 4:**
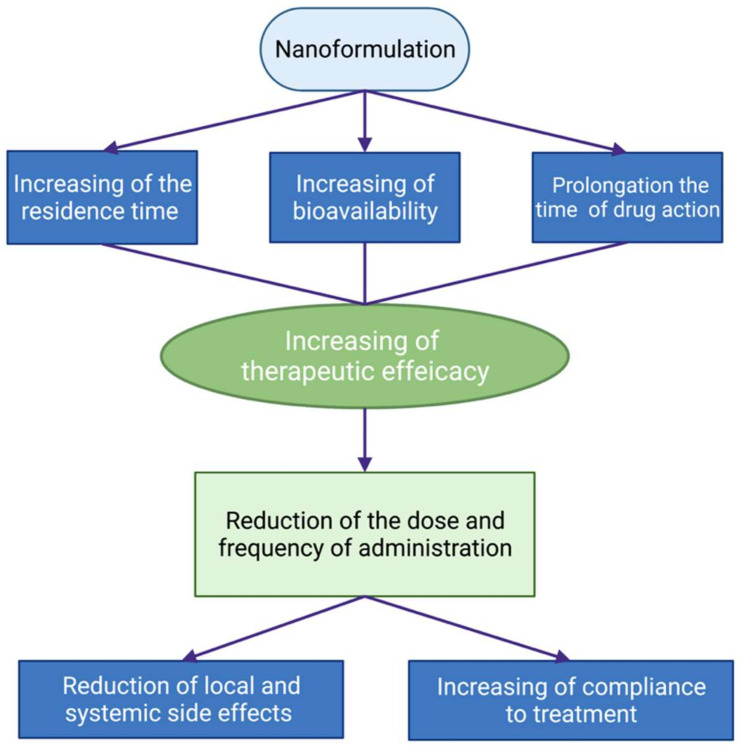
Schematic presentation of the advantages of the use of nanoformulations of ophthalmic drugs. Created with BioRender.com.

Advances in the development of nanotechnology-based approaches to overcome the limits of the topical application of ocular drugs for the therapy of anterior segment eye diseases are quite impressive. The novel formulations were designed for better contact with the eye surface and increased residence time on the eye surface, better penetration into the eye, sustained drug release, and prolonged drug action. So, the effectiveness of these drugs could be substantially increased. Moreover, the dose and frequency of drug administration could be decreased by decreasing the local and systemic side effects of the drug. This is especially important for the treatment of chronic eye diseases, such as glaucoma. The advances in the usage of nanoformulations of drugs in ophthalmology are schematically presented in Figure 4.

Some of these nanoformulations are under clinical trials and some are already on the market, while others are waiting in line for their preclinical studies and trials in clinics. Figure 3 shows some examples of nanoformulations of the drugs at different stages of development.

Thus, nanotechnology demonstrated great potential in the creation of new formulations of ophthalmic drugs. However, future research needs more information on the dosage and administration regimen of these formulations, metabolic ways and ocular toxicity of all formulation components, their pharmacokinetics and pharmacodynamics, the release of the drug in different eye tissues, formulation stability, the influence of the method of the synthesis not only on physico-chemical properties of formulation but also on its physiological effect, the suitability of nanocarriers with respect to biodegradability and patient comfort, etc. It is important to understand and measure the influence of nanoparticulate parameters on their biological effects.

Thus, the perspectives of the use of nanoformulations of ophthalmic drugs look justified and very promising, now meticulous work is required to make it true on a large scale.

## Figures and Tables

**Table 1:** Polymer drug delivery systems target the anterior segment of the eye.

Polymer	Drug	Key Results	Ref.
**Polymeric nanoparticles**			
**Galactosylated chitosan**	Timolol	- Sustained release - Formulation significantly improved the efficacy of the drug in comparison with commercial timolol eye drops - Enhanced bioavailability in comparison with the commercial timolol eye drops	[43]
**Chitosan**	Carteolol	- Sustained release for 24 h - Good transcorneal permeation with non-significant changes in cornea anatomy - Good spread and retention in the precorneal area as compared to the aqueous carteolol solution - Prolonged reduction of IOP ^1^	[44]
**Chitosan, PCL**	Dorzolamide	- 2-fold enhancement in permeation across goat cornea - 3.7-fold higher mucoadhesive strength of nanoparticles compared to the control - Nanoparticles were non-irritant and safe for ocular administration	[45]
**Chitosan**	Ganciclovir	- Significant increase in ganciclovir AUC ^2^ (~4.99-fold) and C_max_ (2.69-fold) in aqueous humor compared to ganciclovir solution - Sustained release	[46]
**Chitosan**	Cyclosporin A	- Cyclosporin A was detected in both aqueous and vitreous humor samples for 72 h - 2-fold of increase in CsA amount incorporated did not influence the ocular penetration of the active agent	[47]
**Chitosan**	Daptomycin	- Encapsulation efficiency ranged from 80 to 97% - Antimicrobial activity of daptomycin was preserved into chitosan nanoparticles	[48]
**Chitosan**	Diclofenac	- Nanoparticles exhibited significantly higher antibacterial activity against *S. aureus* and *B. subtilis*	[49]
**N-Trimethyl Chitosan**	Diclofenac	- Longer retention of the drug in the composition of the particles in an aqueous humor of the rabbits	[50]
**Glycol chitosan**	Dexamethasone	- Progressive release (up to 80%) of the drug in up to 8 h - The drug within particles penetrated the cornea and stayed longer on the corneal surface	[51]
**Chitosan**	Rosmarinic acid	- The nanoparticles were non-cytotoxic to the retinal pigment epithelium (ARPE-19) and the human corneal cell line (HCE-T)	[52]
**Chitosan, sodium alginate**	Azelastine	- Maximum drug entrapment of 73.05% with 65% mucin binding efficiency - Controlled release over the 8-h period - Higher reduction of conjunctival hyperemia and edema	[53]
**Chitosan, sodium alginate + chitosan coating**	5-fluorouracil	- Sustained release - Significantly greater level of the drug in aqueous humor - The enhanced mucoadhesiveness of chitosan-coated chitosan-alginate nanoparticles - C_max_ (24.67 µg/mL) for 5-fluorouracil in nanoparticles was 4-fold higher than 5-fluorouracil solution	[54]
**Chitosan, sodium alginate**	Brimonidine	- The IOP ^1^-lowering effect of the formulation lasted for more than 25 h after a single topical instillation compared with the marketed brimonidine tartrate eye drops	[55]
**Chitosan, sodium alginate**	Betamethasone sodium phosphate	- Sustained release - Encapsulation efficiency was 64% - 84% of the drug was delivered by nanoparticles to the vitreous humor over 5 h and disappeared after 24 h	[56]
**Chitosan, sodium alginate**	Timolol maleate	- No sign of ocular irritation - AUC ^2^ values were found to be 2.27-fold higher for nanoparticle solutions compared to marketed eye drops	[57]
**Albumin, chitosan**	Atropine sulfate	- Superior effects on mydriasis in rabbits than the commercial solutions - Increased bioavailability	[58]
**Chitosan/PEG**	Resveratrol and Quercetin	- Resveratrol in the particles penetrated through the cells of the cornea 6 times higher than the free drug in the ex vivo experiments - Obtained formulations caused a sustained and enhanced decrease in IOP ^1^ (5.5 ± 0.5 mm Hg) of normotensive rabbits	[59]
**Gelatin**		- No serious irritation to the rabbit eyes	[60]
**Gelatin**	Moxifloxacin	- A burst effect in the first hour followed by a controlled release of the drug for the subsequent 12 h - In vivo antibacterial activity of the nanosuspension was more effective against *S. aureus* than the commercially market product - No sign of ocular irritation	[61]
**Gelatin**	Plasmid pMUC5AC	- MUC5AC mRNA and protein were detected in conjunctival cells after in vitro transfection of the nanoparticles - Significantly higher MUC5AC expression in the conjunctiva compared to untreated control and naked plasmid	[62]
**Eudragit RL 100**	Aceclofenac	- 2-fold higher permeation of the drug through excised cornea compared to a marketed aqueous solution of Aceclofenac - No signs of corneal damage - Significantly higher inhibition of polymorphonuclear leukocytes migration and lid closure scores by the nanoparticle formulation - Two-year shelf life at room temperature	[63]
**Eudragit RS 100**	Aceclofenac	- Higher entrapment efficiency of aceclofenac (94.53 ± 1.0%) with prolonged in vitro drug release profiles - Higher transcorneal permeation as compared to aceclofenac aqueous solution - Optimal efficacy of the nanoparticles with significantly higher inhibition of polymorphonuclear leukocytes migration	[64]
**Eudragit RL 100**	Acetazolamide	- The optimum drug concentration at the ocular site for 8 h - Significant IOP ^1^ drop with longer effect	[65]
**Eudragit RL 100**	Tacrolimus	- Long-term stability - Slower elimination with a 1.7-fold higher maximum concentration of Tacrolimus and 5.3-fold higher AUC ^2^ than the aqueous solution	[66]
**PLGA**	Fluorometholone	- Greater anti-inflammatory effects than the commercial formulation - No signs of irritation in the different structures (cornea, iris, and conjunctiva) - Nanoparticles were stable at 25 °C for 15 days	[67]
**PLGA**	Aceclofenac	- 2-fold increase in transcorneal permeation of drug from nanoparticles formulation as compared with an aqueous solution of aceclofenac - No signs of irritation	[68]
**PLGA**	Tacrolimus	- No signs of irritation - Significant increase in Tacrolimus AUC ^2^ (~2.7-fold) and T_max_ (2.33-fold) in aqueous humor compared to tacrolimus solution	[69]
**PLGA**	Tacrolimus	- No signs of eye irritation in rabbits - The superiority of the nanoparticles in retention and permeation into the anterior chamber of the eye compared to the free drug dissolved in oil - A significant decrease in four typical inflammatory markers in a murine model of keratitis, an anterior chamber inflammation - Clinical and histological efficacy in the mainly posterior chamber inflammation model of murine, experimental autoimmune uveitis	[70]
**PLGA**	Pranoprofen	- High encapsulation efficiency 80% - In vivo ocular tolerance and in vivo anti-inflammatory efficacy of nanoparticles in comparison to eye drops conventional dosage form (Oftalar^®^) and free drug solution	[71]
**PLGA**	Xanthohumol	- A significant increase in expression of the transcription factor nuclear factor erythroid 2 - Cytoprotective against oxidative stress in vitro, and significantly reduced ocular surface damage and oxidative stress-associated DNA damage in corneal epithelial cells in the mouse desiccating stress/scopolamine model for dry eye disease in vivo	[72]
**PLGA**	Dorzolamide	- No signs of eye irritation in rabbits - Similar efficacy on IOP ^1^ lowering between one drop of nanoparticles and 4 drops of TRUSOPT^®^	[73]
**PLGA-PEG**	Dexibuprofen	- Enhancing drug retention from nanoparticles and transcorneal permeation - No signs of eye irritation in rabbits - Stability at 25 °C for three months	[74]
**PCL**	Celecoxib	- Drug release was in sustained fashion (<75% drug released after 8 h) - 2-fold higher corneal permeation ex vivo - High anti-inflammatory activity against arachidonic acid-induced ocular inflammation in vivo than marketed formulation	[75]
**Polymeric micelles**			
**PEG-PCL**	Pimecrolimus	- High drug encapsulation capability (97.9%) - Sustained release - High efficacy against Keratoconjunctivitis Sicca	[76]
**PEG-PLA ^3^**	Cyclosporine A	- Cyclosporin A -loaded micellar lyophilized powder was stable for at least 3 months - 4.5-fold increase in retention effect compared with 0.05% Cyclosporin A emulsion	[77]
**PVCL ^4^-PVA ^5^-PEG**	Myricetin	- Enhanced Myricetin’s aqueous solubility and chemical stability - High storage stability - Good in vitro cellular tolerance - Significant improvements in the in vitro antioxidant activity and in vivo anti-inflammatory efficacy against dry eye syndrome	[78]
**PEG-PLA ^3^**	Axitinib	- The area of neovascularization decreased with the use of nanomicelles which confirmed the anti-angiogenic effect of Axitinib-loaded micelles - No signs of eye irritation in rabbits	[79]
**PEG-PCL**	Dexamethasone	- Better inhibitory effect on anterior uveitis in rabbits than the marketed eye drop - The cumulative transcornealpermeation of dexamethasone from the micelles (24.33% in 24 h) was higher than that of the marketed eye drop (20.96% in 24 h)	[80]
**PEG-Polylysine**	Superoxide dismutase	- More effective compared to the free enzyme in decreasing immunogenic uveitis in rabbits - No signs of eye irritation in rabbits	[81]
**Protamine, PEG-b-Polyglutamic acid**	Superoxide dismutase	- High storage stability - Pronounced therapeutic effect without side reactions such as eye irritation - Penetration into anterior eye structures more effectively than the free enzyme	[82]
**Tetronic 701,** **Synperonic PE/F127,** **Synperonic^®^ PE/P84**	Lornoxicam	- A sharp solubility increase from 0.0318 mg/mL up to more than 2.34 mg/mL, representing about a 73-fold increase - The non-irritant nature and good corneal penetrating power of the proposed nano-formulation	[83]

^1^ IOP—Intraocular pressure; ^2^ AUC—Area under the pharmacokinetic curve; ^3^ PLA—poly(lactide); ^4^ PVCL—poly(N-vinylcaprolactam); ^5^ PVA—Polyvinyl acetate.

**Table 2:** Potential ocular drugs under clinical trial and approved by FDA.

Trademark/Drug Name	Drug Molecule	Clinicaltrials.gov Identifier	Status
INVELTYS	Loteprednol etabonate	NCT02163824NCT02793817	FDA ^1^ approved
Bromsite	Bromfenac	NCT01576952	FDA ^1^ approved
Dexasite	Dexamethasone	NCT03192137NCT01543490	Phase III
OCS-01	Dexamethasone	NCT04130802	Phase II
RX-10045	Resolvin E1	NCT02329743	Phase II

^1^ FDA—Federal Drug Agency, USA.

## Data Availability

Not applicable.

## Abbreviations

PLGA	poly(D,L-lactide-co-glycolide)
PCL	poly(ε-caprolactone)
PCA	polycyanoacrylate
PEG	polyethylene glycol
PVP	polyvinylpyrrolidone
AUC	area under the pharmacokinetic curve
IOP	intraocular pressure
PVA	polyvinyl alcohol
PLA	poly(lactide)
PVCL	polyvinyl caprolactam
PEO	poly(ethylene oxide)
Pluronic	(poly(ethylene oxide)-b-poly(propylene oxide)-b-poly(ethylene oxide))
5-FU	5-fluorouracil
SOD	superoxide dismutase
ROS	reactive oxygen species
LCST	lower critical solution temperature
